# Alterations in Brain White Matter Tractography in Older Long-Term Breast Cancer Survivors Treated with Chemotherapy

**DOI:** 10.3390/brainsci16030266

**Published:** 2026-02-27

**Authors:** Ebenezer Daniel, Jonathan R. Young, Frank Deng, Sunita K. Patel, Mina S. Sedrak, Heeyoung Kim, Marianne Razavi, Can-Lan Sun, James C. Root, Tim A. Ahles, William Dale, Bihong T. Chen

**Affiliations:** 1 Department of Diagnostic Radiology, City of Hope National Medical Center, Duarte, CA 91010, USA; ebydaniel89@gmail.com (E.D.);; 2 Department of Population Science, City of Hope National Medical Center, Duarte, CA 91010, USA; 3 Department of Medicine, David Geffen School of Medicine, University of California Los Angeles, Los Angeles, CA 90095, USA; 4 Center for Cancer and Aging, City of Hope National Medical Center, Duarte, CA 91010, USA; 5 Department of Supportive Care Medicine, City of Hope National Medical Center, Duarte, CA 91010, USA; 6 Neurocognitive Research Lab, Memorial Sloan Kettering Cancer Center, New York, NY 10022, USA

**Keywords:** diffusion MRI, diffusion tractography, fiber tracking, DSI studio, chemotherapy, breast cancer, cancer-related cognitive impairment, neuroimaging biomarkers, white matter connectivity

## Abstract

**Purpose:** This study aimed to investigate alterations in brain white matter fiber bundle integrity among older long-term breast cancer survivors treated with chemotherapy, with a focus on identifying potential neural correlates of cancer-related cognitive impairment (CRCI). **Methods:** Women aged 65 years and older were prospectively enrolled and divided into three groups: breast cancer survivors 5 to 15 years after chemotherapy treatment (C+), breast cancer survivors without chemotherapy (C−), and age–sex-matched healthy controls (HC). Participants underwent brain MRI with diffusion tensor imaging and cognitive testing at time point 1 (TP1) upon enrollment and again after two years at time point 2 (TP2). White matter fiber tract integrity was assessed using fractional anisotropy-based (FA) tractography across 80 major fiber bundles in the brain. **Results:** Over the two-year period, both C+ and C− groups exhibited significant reductions in white matter integrity with FA reductions noted in several fiber tracts, including the left inferior fronto-occipital fasciculus (C+ group: *p* < 0.01; C− group: *p* = 0.01), right inferior fronto-occipital fasciculus (*p* < 0.01), left inferior longitudinal fasciculus (C+ group: *p* < 0.01; C− group: *p* = 0.04), right inferior longitudinal fasciculus (C+ group: *p* = 0.04; C− group: *p* = 0.02), right vertical occipital fasciculus (C+ group: *p* < 0.02; C− group: *p* = 0.01), left anterior corticostriatal tracts (C+ group: *p* < 0.01; C− group: *p* = 0.02), right anterior corticostriatal tracts (C+ group: *p* = 0.01; C− group: *p* = 0.02), anterior commissure (C+ group: *p* = 0.01; C− group: *p* = 0.03), and forceps minor (C+ group: *p* = 0.03; C− group: *p* = 0.01). In addition, FA reductions were noted in the left superior longitudinal fasciculus (*p* < 0.01), uncinate fasciculus (*p* = 0.01), thalamic radiation (*p* = 0.04), left optic radiations (*p* = 0.04) and right optic radiations (*p* = 0.03) in the C+ group only. No significant changes over time were detected in the HC group (*p* > 0.05). The fiber tract changes were considered statistically significant at a threshold of *p* < 0.05, with family-wise error (FWE) correction. Significant positive correlation was found between the longitudinal changes in the right inferior fronto-occipital fasciculus and the fluid composite cognition score in the C+ group (R = 0.65 and *p* = 0.03; Pearson’s correlation). **Conclusions:** This study showed continued white matter fiber tract alterations in the older long-term breast cancer survivors who may have cognitive difficulties years after chemotherapy. Diffusion tensor imaging may provide valuable insights into the white matter microstructural correlates of CRCI in older cancer survivors.

## 1. Introduction

Breast cancer is the most often diagnosed cancer among women worldwide, and developments in early detection and treatment have considerably improved survival rates [1,2]. As a result, attention has increasingly shifted toward understanding the long-term effects of cancer and its treatments, particularly in older survivors [3]. One of the most frequently reported and impactful difficulties is cancer-related cognitive impairment (CRCI), which may persist after treatment and can affect various cognitive functions such as memory, attention, executive function, and processing speed [4]. These cognitive declines are especially concerning for older adults, who may be more vulnerable due to age-related neurobiological changes and reduced cognitive reserve [5,6,7,8]. Despite increasing interest, few studies have specifically examined the long-term effects of chemotherapy in older survivors.

Advanced neuroimaging techniques have been instrumental in identifying structural and functional brain changes associated with CRCI and accelerated cognitive aging [9]. Our own previous research has assessed volumetric and morphometric brain alterations in older breast cancer survivors, showing cortical thinning over a two-year longitudinal period in older long-term survivors of breast cancer treated with chemotherapy [10]. In a related study of the same cohort, we investigated cortical folding patterns and found that chemotherapy-treated older survivors exhibited altered gyrification even 5 to 15 years post-treatment [11], which differed in pattern from the age-related decline typically observed in adults of normal aging [12]. These findings are significant, as cortical folding is closely linked to cognitive function, with more complex folding patterns allowing for greater neuronal density and better cognitive functioning in specific brain regions [13,14]. Additionally, our subcortical analysis focusing on the hippocampus, a region critical for memory, neurogenesis, and cognitive aging [15], found bilateral hippocampal volume loss and inward shape deformation in chemotherapy-treated survivors, further supporting the presence of long-term structural brain changes associated with CRCI in older cancer survivors [16].

While gray matter morphometric studies have bestowed valuable understandings of structural brain alterations, white matter fiber tract integrity remains an essential yet underexplored research direction. Diffusion tensor imaging (DTI) offers a complementary perspective to gray matter analysis by assessing axonal integrity and connectivity disruptions in the white matter [17], which are also implicated in CRCI [18]. Previous studies have reported significant reductions in fractional anisotropy (FA), a key DTI metric for white matter integrity in various brain regions post-chemotherapy treatment. For instance, reduced FA has been observed in the superior longitudinal fasciculus and corticospinal tract as early as six months post-treatment in breast cancer patients with a mean age of 49.1 years [19]. Other longitudinal studies have reported white matter changes in frontal, parietal, and occipital regions from pre-treatment to four months post-chemotherapy [20], with partial recovery observed three to four years later [21]. However, persistent FA reductions have also been documented in the cerebral peduncle, inferior fronto-occipital fasciculus, thalamic radiations, and longitudinal fasciculi up to three years post-treatment [22], and white matter microstructural loss has been reported two decades after chemotherapy [23]. Furthermore, our previous study using tract-based spatial statistics (TBSS) revealed significant FA reductions in the anterior corona radiata, the body and genu of the corpus callosum, and the left external capsule in older breast cancer survivors 5 to 15 years post-chemotherapy [24]. These findings have highlighted the impact of chemotherapy on white matter integrity, particularly in regions involved in cognitive processing [25] and interhemispheric communication [26]. TBSS and voxel-based morphometry have been used for white matter assessment, but these methods are methodologically limited in their ability to capture the full complexity of white matter fiber tract architecture and connectivity [27,28]. On the other hand, white matter fiber bundle tractography analysis, which reconstructs specific white matter fiber pathways, offers a more anatomically precise and functionally relevant evaluation of each white matter structure [28,29]. One prior study in younger cancer survivors (ages 30–50 years) more than 12 months post-treatment reported decreased quantitative anisotropy in multiple fiber tracts, including the corticospinal tracts, corpus callosum, fornix, cerebellar tracts, and corticostriatal pathways [30]. Few tractography-based studies in long-term breast cancer survivors have been reported, and the literature is even more limited in older cancer survivors, leaving a gap in knowledge.

In this longitudinal study with a 2-year follow-up assessment, we enrolled older long-term breast cancer survivors with or without chemotherapy and age–sex-matched healthy controls. All study participants received brain MRI scans and cognitive testing. We aimed to build upon our previous research of CRCI in long-term breast cancer survivors by employing automated fiber bundle tractography analysis to assess the structural integrity of major white matter fiber bundles in older long-term breast cancer survivors. Additionally, we assessed correlations between alterations in significantly affected white matter fiber bundles and neurocognitive testing scores. The findings of this study should help to assess neural correlates and potential neuroimaging biomarkers for CRCI in older cancer survivors.

## 2. Methods

### 2.1. Demographic and Clinical Characteristics

This study prospectively enrolled 60 participants at time point 1 (TP1), with 20 individuals assigned to each of the three groups: chemotherapy-treated long-term breast cancer survivors at 5–15 years post-chemotherapy (C+), age–sex-matched long-term breast cancer survivors without chemotherapy (C−), and age–sex-matched healthy controls (HC). However, the study experienced significant attrition over the two-year interval due to various reasons, such as the participants having new memory impairment, participants having a new cancer diagnosis, participant withdrawal, loss to follow-up, and death. In the C+ group, attrition was attributed to 3 participants developing new cancers, 1 experiencing new memory issues, 2 declining further participation, 1 relocating out of state, and 1 passing away. In the C− group, 5 participants were lost to follow-up, 2 declined to continue, and 1 relocated out of state. The HC group experienced attrition due to 3 participants being lost to follow-up and 2 relocating out of state. Consequently, the final cohort with both TP1 and time point 2 (TP2) consisted of 12 participants in each of the C+ and C− groups, and 15 participants in the HC group.

This neuroimaging sub-study was conducted as part of a larger multi-center clinical trial assessing brain structure and function in older long-term breast cancer survivors (parent trial: Cognition in Older Breast Cancer Survivors: Treatment Exposure, APOE and Smoking History, NCT02122107). The study was carried out following the principles of the Declaration of Helsinki and received approval from the Institutional Review Board at City of Hope National Medical Center (IRB# 14283; approval date: 1 December 2014). The parent trial has been registered on ClinicalTrials.gov (NCT02122107), and all participants provided written informed consent to the study.

### 2.2. Neuropsychological Testing

Cognitive function was assessed using the NIH Toolbox Cognition Battery [31,32]. This battery yielded seven domain-level measures, each capturing a distinct aspect of cognitive functioning: picture sequence memory, pattern comparison processing speed, picture vocabulary, oral reading recognition, list sorting working memory, flanker inhibitory control, and dimensional change card sorting. Furthermore, three composite scores were calculated for crystallized intelligence, fluid composite cognition, and overall cognitive function.

A generalized linear model (GLM) was employed to account for repeated measures within subjects. The model included group (C+, C−, HC) and time point (TP1, TP2) as categorical fixed effects. Three primary analyses were conducted using GLM: (1) comparison of neuropsychological scores between groups at TP1, (2) evaluation of within-group changes over time, and (3) assessment of group-by-time interaction effects. All statistical analyses were performed using SAS version 9.3 (SAS Institute, Cary, NC, USA). Detailed findings related to neuropsychological performance for this cohort have been previously reported in our study examining cortical thickness [10].

### 2.3. DTI Data Acquisition

In this study, brain MRI scans were acquired on the same Siemens Verio 3T scanner (Erlangen, Germany). The DTI data were collected using the following parameters: 20 diffusion directions, a repetition time (TR) of 9400 milliseconds (ms), an echo time (TE) of 87 ms, a field of view (FOV) of 224 × 224 mm^2^, a flip angle of 90°, voxel dimensions of 1.75 × 1.75 × 2.34 mm^3^, 64 slices, and a b-value of 1000 s/mm^2^. The detailed DTI data acquisition was previously reported in our published paper on a TBSS study [24].

### 2.4. Automated Fiber Tractography Analysis

DTI data were preprocessed and reconstructed using the DSI Studio software (version 2025) (https://dsi-studio.labsolver.org/download.html, date of access (10 September 2023)) [33,34,35], specifically employing the ‘Step T1’ and ‘Step T2’ processes within the tractography module. For each group at TP1, a FA connectometry database was created using the aggregated data via ‘Step C1’. For longitudinal analysis, the data were organized using the “longitudinal scan” option, followed by the “longitudinal difference” function to update the tractography database accordingly. Fiber tracking and visualization were conducted using ‘Step T3’ in DSI Studio. During this step, we applied fine-tuned tracking parameters, selecting tracts with lengths ranging from 30 to 300 mm and setting a termination threshold at 5000 tracts. Statistical analysis was performed on the resulting tracts to compute mean FA values. These values were then used to assess statistical differences between groups, within-group changes over time, and to conduct correlation analysis. All statistical tests were carried out using IBM SPSS Statistics (Version 29, IBM Corp., Chicago, IL, USA). Group comparisons at TP1 (C+ vs. C−, C+ vs. HC, and C− vs. HC) were evaluated using independent-sample *t*-tests. Paired *t*-tests were used to assess within-group longitudinal changes over the two-year interval. Additionally, a GLM was employed to examine group-by-time interaction effects across the three groups.

### 2.5. Correlation Analysis

To explore the relationship between white matter fiber tract integrity and cognitive performance, we conducted correlation analyses between the mean FA values of fiber tracts (derived from DSI Studio) and the three neuropsychological composite scores, i.e., crystallized, fluid, and total composite scores obtained from the NIH Toolbox testing at TP1. For longitudinal analysis, we assessed the association between the changes in FA values and the changes in NIH Toolbox composite scores between TP1 and TP2. All correlation analyses were performed using the Bivariate Correlation method in IBM SPSS Statistics (Version 29), with statistical significance determined at a *p*-value threshold of 0.05.

## 3. Results

### 3.1. Clinical and Demographic Characteristics

For cancer survivors, the time from diagnosis to TP1 was recorded. The mean (±SD) duration for the C+ group was 7.42 ± 2.34 years, and it was 8.20 ± 2.86 years for the C− group, with no statistically significant difference between the two groups (*p* = 0.372). Additionally, the mean time from chemotherapy completion to TP1 in the C+ group was 6.52 ± 2.33 years. As shown in Table 1, there were no significant differences among the groups in terms of age (*p* = 0.75), education level (*p* = 0.80), or racial distribution (*p* = 0.37). Detailed demographic and clinical data have been previously published in our study on the same cohort focusing on cortical thickness [10].

At TP1, 15 of the 20 patients in the C+ group and 14 of the 20 patients in the C− group received endocrine therapy, and there was no significant difference in the number of participants with endocrine therapy between the two cancer groups (*p* = 0.999). Among the patients with data available for both TP1 and TP2, eight patients in the C+ group and seven in the C− group received endocrine therapy, also showing no significant difference in the number of participants with endocrine therapy between the two cancer groups (*p* = 0.319). Chemotherapy regimen administered to the patients in the C+ group included the following: ddAC-T (dose-dense doxorubicin (Adriamycin) and cyclophosphamide, followed by paclitaxel (Taxol)), AC-T (doxorubicin hydrochloride (Adriamycin) and cyclophosphamide, followed by paclitaxel (Taxol)), TC (docetaxel and cyclophosphamide), AC (doxorubicin (Adriamycin) and cyclophosphamide), CMF (cyclophosphamide, methotrexate, and 5-fluorouracil (5-FU)), AC T+H (doxorubicin hydrochloride (Adriamycin) and cyclophosphamide, followed by paclitaxel (Taxol) and trastuzumab (Herceptin)), and TCH (docetaxel, carboplatin, and trastuzumab (Herceptin)). In the C+ group, most participants received the TC regimen (docetaxel + cyclophosphamide), accounting for nine individuals (75%). One participant each (8% per regimen) received the AC regimen (doxorubicin + cyclophosphamide), the CMF regimen (cyclophosphamide, methotrexate, and 5-fluorouracil), and the TAC regimen (docetaxel, doxorubicin, and cyclophosphamide).

### 3.2. Automated White Matter Fiber Tractography

**Figure 1:**
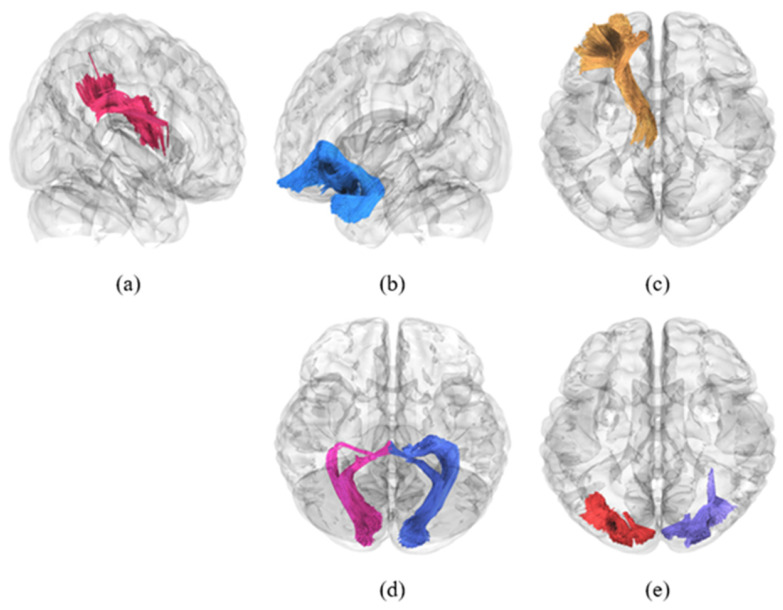
Representative white matter tractography showing fiber bundles with a significant longitudinal decrease in fractional anisotropy within the chemotherapy group (C+). (**a**) Superior longitudinal fasciculus (L), (**b**) uncinate fasciculus (L), (**c**) anterior thalamic radiation (L), (**d**) bilateral optic radiation, and (**e**) bilateral vertical occipital fasciculus. The left vertical occipital fasciculus shows marginal significance in the C+ group at a *p*-value threshold of 0.05, while the right vertical occipital fasciculus shows a significant decrease in both cancer groups (C+ and C−). Abbreviations: L, left; R, right.

**Figure 2:**
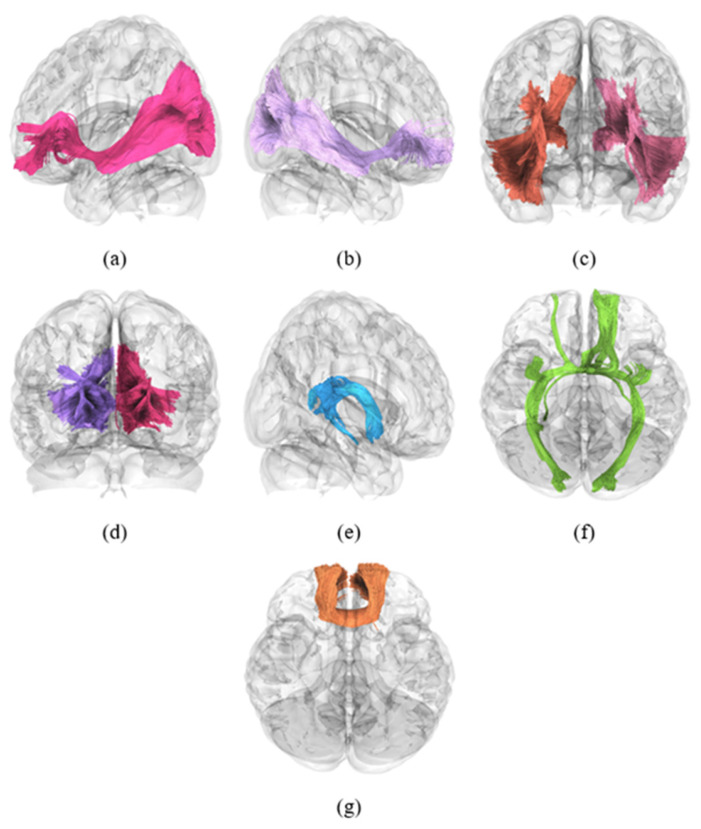
Representative white matter tractography showing fiber bundles with significant longitudinal decreases in fractional anisotropy within the two cancer groups (C+ and C−). (**a**) Inferior fronto-occipital fasciculus (L), (**b**) inferior fronto-occipital fasciculus (R), (**c**) bilateral inferior longitudinal fasciculus, (**d**) bilateral anterior corticostriatal tract, (**e**) fornix (L), (**f**) anterior commissure, and (**g**) corpus callosum forceps minor. Abbreviations: C+, chemotherapy group; C−, non-chemotherapy control group; L, left; R, right.

**Figure 3:**
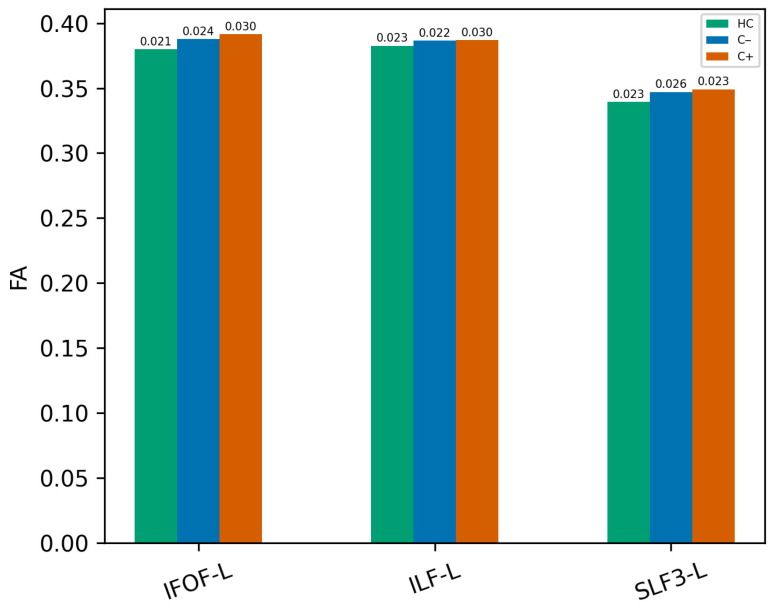
Fractional anisotropy values at time point 1 (TP1) in representative white matter tracts across the chemotherapy group (C+), the non-chemotherapy group (C−), and the healthy control (HC) group and standard deviation values are indicated above each bar. Abbreviations: IFOF, inferior fronto-occipital fasciculus; ILF, inferior longitudinal fasciculus; SLF-3 superior longitudinal fasciculus 3; L, left.

**Figure 4:**
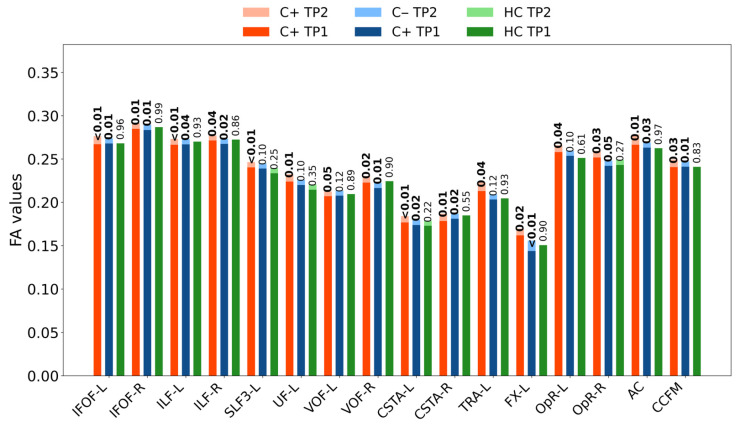
Longitudinal changes in fractional anisotropy (FA) values from time point 1 (TP1) to time point 2 (TP2) in all 16 significant white matter fiber tracts across the chemotherapy group (C+), the non-chemotherapy group (C−), and the healthy control (HC) group. Solid bars indicate FA at TP1, lighter shaded bars indicate FA at TP2, and *p*-values for changes between TP1 and TP2 are displayed above each bar pair; *p* values <0.05 are shown in bold. Fiber tracts shown here: (a) IFOF-L, (b) IFOF-R, (c) ILF-L, (d) ILF-R, (e) SLF3-L, (f) UF-L, (g) VOF-L, (h) VOF-R, (i) CSTA-L, (j) CSTA-R, (k) TRA-L, (l) FX-L, (m) OpR-L, (n) OpR-R, (o) AC, (p) CCFM. Abbreviations: IFOF, inferior fronto-occipital fasciculus; ILF, inferior longitudinal fasciculus; SLF3, superior longitudinal fasciculus3; UF, uncinate fasciculus; VOF, vertical occipital fasciculus; CSTA, anterior corticostriatal tract; TRA, anterior thalamic radiation; FX, fornix; OpR, optic radiation; AC, anterior commissure; CCFM, corpus callosum forceps minor; L, left; R, right.

Automated FA-based white matter tractography analysis was performed using DSI Studio, focusing on 80 major white matter tracts. At TP1, no significant differences were observed in white matter fiber tract integrity between the groups (C+ vs. C−, C+ vs. HC, and C− vs. HC, all *p* > 0.05). Longitudinal within-group analysis revealed significant reductions in white matter tract integrity in the C+ group. Specifically, decreased FA values were observed in the bilateral inferior fronto-occipital fasciculus, bilateral inferior longitudinal fasciculus, left superior longitudinal fasciculus III, left uncinate fasciculus, bilateral vertical occipital fasciculus, bilateral anterior corticostriatal tracts, left anterior thalamic radiation, left fornix, bilateral optic radiations, anterior commissure, and forceps minor of the corpus callosum (Figure 1 and Table 2, all *p* < 0.05). In the C− group, significant longitudinal within-group reductions were noted in the bilateral inferior fronto-occipital fasciculus, bilateral inferior longitudinal fasciculus, right vertical occipital fasciculus, bilateral anterior corticostriatal tracts, left fornix, anterior commissure, and corpus callosum forceps minor (Figure 2 and Table 2, all *p* < 0.05). Of the 16 tracts analyzed, three representative tracts are shown as examples in Figure 3, while the longitudinal changes across all 16 tracts are presented in Figure 4. No significant longitudinal changes were detected in the HC group (*p* > 0.05). However, no significant group-by-time interaction effects were observed across the three groups. All reported findings were statistically significant at a threshold of *p* < 0.05, corrected for family-wise error (FWE).

### 3.3. Neuropsychological Testing Data

At TP1, the C+ group performed significantly worse than the C− group on the crystallized composite score (*p* = 0.04) and oral reading recognition (*p* = 0.02). In the longitudinal analysis within the C+ group, there were significant declines in the total composite score (*p* = 0.01), fluid composite cognition (*p* = 0.03), and picture vocabulary (*p* = 0.04). Additional decrease approached statistical significance for the crystallized composite score (*p* = 0.057) and picture sequence memory (*p* = 0.05). No significant changes over time were observed in either the C− or HC groups. Also, group-by-time interaction effects were not significant across the three groups (*p* > 0.05).

### 3.4. Correlation Analysis

In the C+ group, a significant positive correlation was identified between longitudinal changes in fluid composite cognition scores and alterations in the right inferior fronto-occipital fasciculus (*p* = 0.03, R = 0.65). No significant correlations were observed in the C− or HC group (Table 3).

## 4. Discussion

In this study, we identified diminished white matter fiber bundle integrity in older long-term breast cancer survivors with and without exposure to chemotherapy at 5 to 15 years post-treatment. Additionally, we observed a significant positive correlation between the longitudinal changes in white matter tracts and the changes in cognitive performance over the two-year follow-up interval. To our knowledge, this was the first prospective longitudinal study assessing the long-term multifactorial effects including cancer-treatment-related factors on white matter tractography in older breast cancer survivors.

This study focusing on white matter tractography alterations extended and complemented our prior TBSS study on the same cohort, which showed reduced white matter integrity in the left anterior corona radiata, the body and genu of the corpus callosum, and the left external capsule [24]. In this study, we observed reduced white matter fiber tract integrity in several key tracts traversing and intersecting these previously reported white matter regions using TBSS methods in older breast cancer survivors, including the anterior corticostriatal tract, thalamic radiation, and uncinate fasciculus. These tract-level white matter disruptions provided a structural basis for the voxel-level changes previously reported, suggesting that the TBSS-identified regions may exhibit broader disruptions of the interconnected white matter pathways [36]. Previous studies have employed TBSS to detect voxel-wise alterations in white matter using skeletonized diffusion maps [37]. While TBSS is effective for identifying widespread white matter microstructural changes, the tractography-based approach used in this study offered the advantage of reconstructing and visualizing specific white matter pathways in three dimensions [30]. The tractography approach for this study enabled a more anatomically precise fiber bundle analysis of structural changes over time and between groups [38].

The anterior segment of the corticostriatal pathway, which connects the frontal cortex to the caudate nucleus, plays a critical role in executive functions such as decision-making, planning, goal-directed behavior, and working memory [39,40]. Our findings were consistent with previous studies that reported bilateral corticostriatal alterations in younger breast cancer patients aged 30–50 years, approximately one-year post-treatment [30]. However, our study differed from the prior study in several aspects, i.e., our focus on an older population (aged 65 years and older) and on long-term effects in cancer survivor (5–15 years post-chemotherapy) and a longitudinal study design and follow-up data over a 2-year interval. While the earlier study reported widespread corticostriatal disruptions, our findings were more localized to the anterior portion of this fiber tract. This finding suggested partial recovery of the fiber tract over time, with persistent alterations only in the anterior segment. Additionally, we observed changes in corticostriatal tracts in both cancer groups (the C+ and C− groups), regardless of chemotherapy exposure, implicating a multifactorial effect including cancer diagnosis and cancer treated-related factors, either chemotherapy or endocrine therapy, on white matter fiber tract integrity.

In the C+ group, we also observed alterations in the third segment of the superior longitudinal fasciculus, a tract connecting the supramarginal gyrus with the ventral premotor and prefrontal cortices. This white matter fiber tract is known to support language processing, complex motor planning, and visuospatial attention [41]. Although this tract was not among the regions previously identified in our TBSS analysis, its involvement in the present tractography study may represent additional long-range white matter disruption that was not captured by voxel-wise methods such as TBSS [24]. These tractography-based changes were only noted in the C+ group and may reflect broader tract-level alterations associated with chemotherapy exposure in older cancer survivors.

This study identified changes in the forceps minor and anterior commissure through tractography, indicating potential disruptions in interhemispheric communication between the brain regions in the frontal and temporal lobes. The forceps minor traverses the genu of the corpus callosum, connects the bilateral prefrontal cortices and supports executive functions [42]. The anterior commissure links the temporal lobes and is involved in emotional regulation and memory processing [42,43]. These white matter tracts, along with the genu and body of the corpus callosum—which connect prefrontal, sensorimotor, and parietal cortices—form a core commissural network essential for cognitive and emotional integration. Notably, our previous TBSS study revealed reduced integrity specifically in the genu and body of the corpus callosum in this cohort [24]. The present tractography analysis provided a more detailed, pathway-specific visualization of these disruptions, highlighting their role in interhemispheric information transfer and their potential contribution to cognitive and emotional dysfunction in the older long-term cancer survivors.

We also noted white matter loss in the left inferior fronto-occipital fasciculus and uncinate fasciculus, reflecting disruptions in long-range associative fiber tracts. The inferior fronto-occipital fasciculus connects the frontal, temporal, parietal, and occipital lobes and is involved in semantic processing, attention, and visual integration [44,45,46]. The uncinate fasciculus links the orbitofrontal cortex with the anterior temporal lobes and plays a role in memory and emotional regulation [47,48]. Both fiber bundle pathways are close to the external capsule [49], where our previous TBSS analysis showed reduced white matter integrity [24]. This anatomical overlap suggested that the disruptions in the inferior fronto-occipital fasciculus, uncinate fasciculus and external capsule may contribute to CRCI in the older cancer survivors.

We also found a significant positive correlation between longitudinal changes in the right inferior fronto-occipital fasciculus and the changes in the fluid composite cognition testing score in the C+ group. Although both cancer groups (C+ and C−) showed FA reductions in inferior fronto-occipital fasciculus, only the C+ group demonstrated an association with cognitive decline based on the neuropsychological testing score, suggesting a potential chemotherapy-specific treatment effect. These findings were consistent with prior studies reporting reduced FA in the inferior fronto-occipital fasciculus up to three years post-chemotherapy [22] and over nine years after treatment [50]. However, the present study showed a similar pattern of white matter changes over time in both the C+ and C− survivors, with 11 tracts affected in both cancer groups, while the HC group showed no changes over time. This pattern of white matter fiber tract changes was consistent with our previous findings of no significant differences between the C+ and C− groups on neuropsychological tests but both cancer groups performed worse than the HC group, supporting the accelerated cognitive aging hypothesis in older cancer survivors [51]. These results also implicated that survivorship-related factors, regardless of treatment type (with or without chemotherapy exposure), may underlie the structural and cognitive changes seen in the cancer groups.

This study had several limitations. First, the sample size was modest, and a considerable number of participants dropped out of the study across the two-year follow-up period. Second, the cohort primarily consisted of older white women, which may limit the generalizability of the findings to younger patients or patients of other races and ethnicity. Future studies should include more diverse populations. Third, while tractography provided valuable insights into white matter fiber tract integrity, it was limited by sensitivity to crossing fibers and partial volume averaging effects [52]. Advanced diffusion models and multimodal neuroimaging strategy may help address these limitations. Lastly, the major difference in stage distribution in the cancer groups, i.e., 75% of the C+ group being stage II/III and 85% of the C− group being DCIS/stage I at TP1, was an inherent limitation of this study. It was challenging to recruit patients with high-stage breast cancer but without chemotherapy since chemotherapy is a recommended treatment strategy for stage II/III breast cancer. On the other hand, patients with lower stages such as DCIS/I were more likely to be treated with endocrine therapy rather than chemotherapy per National Comprehensive Cancer Network (NCCN) guidelines for breast cancer treatment [53]. This study was also limited by the lack of proper control for other potential confounders such as treatment history, treatment intensity, recurrence risk, comorbidities, psychosocial factors, unstated emotional stress, cardiovascular risk factors and white matter hyperintensities in the older cancer survivors. Despite these limitations, this study contributed novel information to CRCI research focusing on alterations in white matter tractography in older long-term breast cancer survivors.

## 5. Conclusions

This longitudinal white matter tractography study revealed reductions in fiber bundle integrity in the older long-term breast cancer survivors, which was correlated with cognitive function. These findings implicated alterations in white matter tractography may potentially be the neural correlates of CRCI in older cancer survivors. Future study with larger, more diverse cohorts is warranted to validate these findings and to further elucidate the neurobiological mechanisms of cognitive functioning in older cancer survivors.

## Figures and Tables

**Table 1:** Clinical and demographic information of the study cohort.

	TP1				Longitudinal Subset Analyzed in This Study (with Both TP1 and TP2 Available)			
Parameters	C+ N = 20	C− N = 20	HC N = 20	*p*	C+ N = 12	C− N = 12	HC N = 15	*p*
**Age** (years)								
Mean (SD)	73.5 (5.06)	76.85 (4.63)	74.00 (6.09)	0.106	73.75 (5.41)	76.50 (4.28)	74.53 (6.73)	0.48
Range	66–84	69–86	66–88		68–84	71–86	66–88	
**Race (N, %)**								
Caucasian	15 (75)	18 (90)	18 (90)	0.096	10 (83)	11 (92)	14 (93)	0.76
Black	1 (5)	2 (10)	-		1 (8)	1 (8)	-	
Other	4 (20)	-	2 (20)		1 (8)	-	1 (7)	
**Education** (N, %)								
High school or less	4 (20)	5 (25)	6 (30)	0.359	3 (25)	4 (33)	4 (27)	0.99
Above high school	16 (80)	15 (75)	14 (70)		9 (75)	8 (67)	11 (73)	
**AJCC Stage** (N, %)								
DCIS/I	5 (25)	17 (85)	.		2 (17)	10 (83)		
II/III	15 (75)	3(15)	.		10 (83)	2 (17)		

Note: Statistical significance set at a threshold of *p* < 0.05. Abbreviations: C+, chemotherapy group; C−, non-chemotherapy group; HC, healthy control group; TP1, time point 1; TP2, time point 2; AJCC = American Joint Committee on Cancer; DCIS = Ductal Carcinoma in Situ; N = number of participants.

**Table 2:** Longitudinal within-group alterations in white matter fiber tracts based on fractional anisotropy diffusion metrics.

Tract Name	C+ Group			C− Group			HC Group		
	*Mean*	*Std*	*p Value*	*Mean*	*Std*	*p Value*	*Mean*	*Std*	*p Value*
Inferior fronto-occipital fasciculus (L)	0.02	0.02	**<0.01**	0.01	0.01	**0.01**	0.00	0.03	0.96
Inferior fronto-occipital fasciculus (R)	0.01	0.01	**0.01**	0.01	0.01	**0.01**	0.00	0.02	0.99
Inferior longitudinal fasciculus (L)	0.01	0.01	**<0.01**	0.01	0.02	**0.04**	0.00	0.02	0.93
Inferior longitudinal fasciculus (R)	0.01	0.01	**0.04**	0.01	0.01	**0.02**	0.00	0.02	0.86
Superior longitudinal fasciculus3 (L)	0.01	0.01	**<0.01**	0.01	0.02	0.10	0.01	0.02	0.25
Uncinate fasciculus (L)	0.01	0.01	**0.01**	0.01	0.02	0.10	0.01	0.04	0.35
Vertical occipital fasciculus (L)	0.01	0.01	*0.05*	0.01	0.03	0.12	0.00	0.02	0.89
Vertical occipital fasciculus (R)	0.01	0.01	**0.02**	0.01	0.01	**0.01**	0.00	0.02	0.90
Corticostriatal tract anterior (L)	0.01	0.01	**<0.01**	0.01	0.01	**0.02**	0.01	0.02	0.22
Corticostriatal tract anterior (R)	0.01	0.01	**0.01**	0.01	0.01	**0.02**	0.00	0.02	0.55
Thalamic radiation anterior (L)	0.02	0.02	**0.04**	0.01	0.03	0.12	0.00	0.03	0.93
Fornix (L)	0.01	0.02	**0.02**	0.02	0.01	**<0.01**	0.00	0.02	0.90
Optic radiation (L)	0.01	0.02	**0.04**	0.01	0.01	0.10	0.00	0.03	0.61
Optic radiation (R)	0.01	0.02	**0.03**	0.01	0.02	*0.05*	0.01	0.03	0.27
Anterior commissure	0.02	0.02	**0.01**	0.01	0.01	**0.03**	0.00	0.03	0.97
Corpus callosum forceps minor	0.01	0.02	**0.03**	0.01	0.02	**0.01**	0.00	0.04	0.83

Note: The numbers in the table correspond to the mean differences in fractional anisotropy value ± standard deviations, with the associated *p*-values. Abbreviations: C+: chemotherapy, C−: no-chemotherapy group, HC: healthy control, L: Left, R: hemisphere, Std: Standard deviation. Significant *p*-value set at a threshold of 0.05.

**Table 3:** Correlation of longitudinal changes between the right inferior fronto-occipital fasciculus white matter tract and the fluid composite cognition score.

	C+ Group	C− Group	HC Group
R	0.65	−0.37	0.42
*p*-value	***0.03***	0.27	0.12

Abbreviations: C+ = chemotherapy group; C− = non-chemotherapy group; and HC = healthy control group. R is the Pearson’s correlation coefficient with significance at *p* < 0.05.

## Data Availability

The data are not publicly available due to privacy and ethical restrictions.
